# Supplementary material for: Is Citicoline Effective in Preventing and Slowing Down Dementia?—A Systematic Review and a Meta-Analysis
Source: Nutrients. 2023 Jan 12;15(2):386. doi: 10.3390/nu15020386 (PMC9866349; doi:10.3390/nu15020386)
Supplement: Supplementary file 1 [file nutrients-15-00386-s001.zip › nutrients-2127932-supplementary.pdf]

SUPPLEMENTARY MATERIAL

Table S1. search strings.

((("Alzheimer Disease"[Mesh]) OR “Mild cognitive impairment"[Mesh]) AND "Choline"[Mesh]) OR "Cytidine Diphosphate Choline"[Mesh]
((("Alzheimer Disease"[Mesh]) OR "Cognitive Dysfunction"[Mesh]) AND "Choline"[Mesh]) OR "Cytidine Diphosphate Choline"[Mesh]
((("Alzheimer Disease"[Mesh]) OR "Cognitive Decline"[Mesh]) AND "Choline"[Mesh]) OR "Cytidine Diphosphate Choline"[Mesh]
((("Alzheimer Disease"[Mesh]) OR "Cognitive Decline"[Mesh]) AND “Citicoline"[Mesh])
((("Alzheimer Disease"[Mesh]) OR "Cognitive Dysfunction"[Mesh]) AND “Citicoline"[Mesh])
((("Alzheimer Disease"[Mesh]) OR “Mild cognitive impairment"[Mesh]) AND “Citicoline"[Mesh])
((("Vascular Dementia"[Mesh]) OR "Cognitive Decline"[Mesh]) AND “Citicoline"[Mesh])
((("Vascular Dementia"[Mesh]) OR "Cognitive Dysfunction"[Mesh]) AND “Citicoline"[Mesh])
((("Vascular Dementia"[Mesh]) OR “Mild cognitive impairment"[Mesh]) AND “Citicoline"[Mesh])
(Alzheimer[ti] OR "mild cognitive impairment"[ti]) AND choline OR citicoline AND 2010:2022[dp]
(Alzheimer[ti] OR “cognitive dysfunction"[ti]) AND choline OR citicoline AND 2010:2022[dp]
(Alzheimer[ti] OR “cognitive decline"[ti]) AND choline OR citicoline AND 2010:2022[dp]
(Vascular dementia[ti] OR “cognitive decline"[ti]) AND choline OR citicoline AND 2010:2022[dp]
(Vascular dementia[ti] OR “cognitive dysfunction"[ti]) AND choline OR citicoline AND 2010:2022[dp]
(Vascular dementia[ti] OR "mild cognitive impairment"[ti]) AND choline OR citicoline AND 2010:2022[dp]

FUNNEL PLOTS

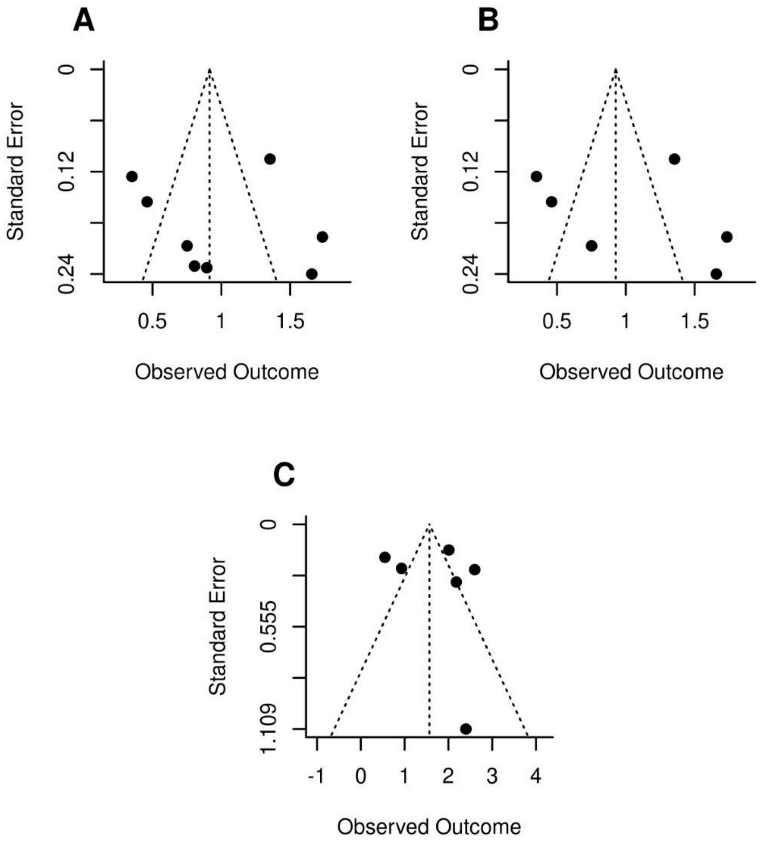

Figure S1: Funnel plot of all studies (panel A), only observational studies (panel B), and only studies using MMSE as outcome measure (panel C). SD were imputed assuming a large correlation coefficient (r = 0.9).

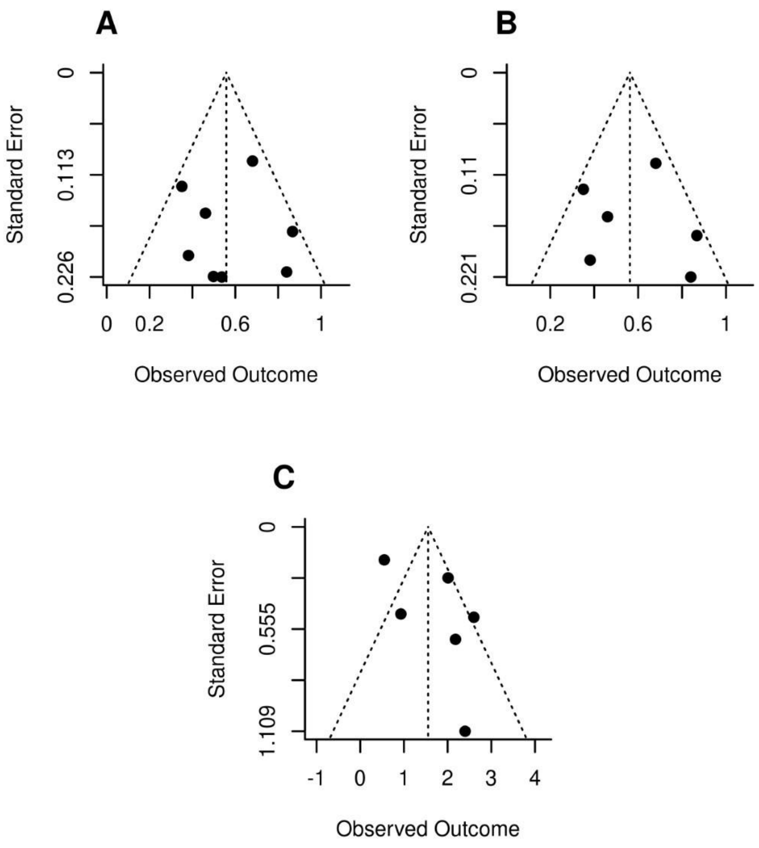

Figure S2: Funnel plot of all studies (panel A), only observational studies (panel B), and only studies using MMSE as outcome measure (panel C). SD were imputed assuming a small correlation coefficient (r = 0.6).
